# Deubiquitinating enzymes (DUBs): DoUBle-edged swords in CNS autoimmunity

**DOI:** 10.1186/s12974-020-01783-8

**Published:** 2020-04-06

**Authors:** Jing Ruan, Dirk Schlüter, Xu Wang

**Affiliations:** 1 Department of Pathology, The First Affiliated Hospital of Wenzhou Medical University, Wenzhou, 325000 China; 2 Institute of Medical Microbiology and Hospital Epidemiology, Hannover Medical School, Carl-Neuberg-Straße-1, 30626 Hannover, Germany; 3 Cluster of Excellence RESIST (EXC 2155), Hannover Medical School, 30625 Hannover, Germany; 4 Chemical Biology Research Center, School of Pharmaceutical Sciences, Wenzhou Medical University, Chashan High Education Park, Wenzhou, 325035 China

**Keywords:** CNS autoimmunity, Multiple sclerosis, Experimental autoimmune encephalomyelitis, Ubiquitination, Deubiquitinating enzymes

## Abstract

Multiple sclerosis (MS) is the most common autoimmune disease of the CNS. The etiology of MS is still unclear but it is widely recognized that both genetic and environmental factors contribute to its pathogenesis. Immune signaling and responses are critically regulated by ubiquitination, a posttranslational modification that is promoted by ubiquitinating enzymes and inhibited by deubiquitinating enzymes (DUBs). Genome-wide association studies (GWASs) identified that polymorphisms in or in the vicinity of two human DUB genes *TNFAIP3* and *USP18* were associated with MS susceptibility. Studies with experimental autoimmune encephalomyelitis (EAE), an animal model of MS, have provided biological rationale for the correlation between these DUBs and MS. Additional studies have shown that other DUBs are also involved in EAE by controlling distinct cell populations. Therefore, DUBs are emerging as crucial regulators of MS/EAE and might become potential therapeutic targets for the clinical treatment of MS.

## Background

Intracellular signaling pathways control virtually all cellular functions ranging from homeostasis to cellular stress responses. Signal transduction is regulated by specific and orchestrated post-translational modifications (PTMs) including phosphorylation, SUMOylation, neddylation, ISGylation, acetylation, methylation, succinylation, and ubiquitination [1–4]. With few exceptions like citrullination and deamination, PTMs are usually rapid and reversible, ensuring a quick and economical adaptation to micro-environmental changes. Ubiquitination, also known as ubiquitylation, modifies target proteins by adding one or more ubiquitins, which are evolutionarily conserved 76-aa small molecules [5]. Ubiquitination is catalyzed by a cascade of three ubiquitin-modifying enzymes: ubiquitin-activating enzymes (E1s, 2 members in humans), ubiquitin-conjugating enzymes (E2s, ~ 40 members), and ubiquitin-ligases (E3s, ~ 600 members). In the initiating step, ubiquitin is activated by an E1 in the presence of ATP, forming a thioester bond between the C-terminal glycine of ubiquitin and the cysteine sulfhydryl group of E1. Subsequently, the activated ubiquitin is transferred to the cysteine residue in the active site of an E2, which also determines the type of substrate ubiquitination. In the final step, ubiquitin is covalently attached to a substrate and this is coordinated by a specific E3, which defines substrate specificity [6]. The three-step process enables the quick and efficient ubiquitination of the target substrates, conferring the cells a swift adaptation to changes within minutes. Ubiquitination of a substrate can be mediated by a single ubiquitin molecule (monoubiquitination), several single ubiquitin molecules (multi-monoubiquitination or poly-monoubiquitination), or polyubiquitin chains (polyubiquitination) [7]. The polyubiquitin chains contain several ubiquitin molecules, which are covalently linked by an N-terminal methionine residue (Met1, linear linkage) or one of the seven lysine residues, i.e., Lys6, Lys11, Lys27, Lys29, Lys33, Lys48, and Lys63 [8]. The type of ubiquitination determines the fate of target substrates as well as cellular functions. Ubiquitination was initially identified as a mechanism regulating protein degradation. Proteins conjugated with Lys48-linked ubiquitination are destined for degradation via the ubiquitin-proteasome system (UPS). In addition to Lys48-specific ubiquitination, protein degradation can also be triggered by Lys6-, Lys11-, Lys27-, Lys29-, and Lys33-linked polyubiquitination [7]. However, the function of ubiquitination is not restricted to protein degradation, and non-degradative activities can also be regulated by ubiquitination [9]. For example, monoubiquitination and multi-monoubiquitination regulates membrane trafficking, endocytosis, and viral budding while linear (Met1) and Lys63-linked ubiquitination has been proved to be critical in intracellular signal transduction. Ubiquitination has been implicated in the regulation of a broad range of cellular processes, including autophagy, cell cycle regulation, DNA repair, cell death, as well as initiation and regulation of both the innate and adaptive immunity [10].

Ubiquitination is a highly dynamic and reversible process and can be counter-regulated by deubiquitinating enzymes (DUBs), which reduce ubiquitination by either directly removing ubiquitin(s) from target proteins or blocking the formation of ubiquitin chains [8]. To date, around 100 DUBs have been identified in humans and, based on structures, they are categorized into seven families: the ubiquitin-specific proteases (USPs), the ovarian tumor proteases (OTUs), the JAB1/MPN/MOV34 metalloproteases (JAMMs), the ubiquitin C-terminal hydrolases (UCHs), the Josephins, the motif interacting with ubiquitin (MIU)-containing novel DUB family (MINDYs), and ZUP1 [11]. Except for JAMMs, which are metalloproteinases, all DUBs are classified as cysteine proteases.

Autoimmunity results from aberrant immune reaction against self-antigens, leading to chronic inflammation and tissue destruction. Multiple sclerosis (MS) is a chronic inflammatory demyelinating disease and one of the most prevalent autoimmune disease of the central nervous system (CNS), which is characterized by the invasion of macrophages, T cells, and B cells in the CNS as well as immune-induced demyelination, neural damage, and plaque formation [12]. Symptoms of MS include visual disturbances, paresthesias, muscle weakness, and ataxia, and it is the leading cause of disability resulting from CNS inflammation in young adults in western countries [13]. The clinical course of MS is variable, and the disease may present with relapsing-remitting, primary and secondary progressive symptoms. The cause of MS is poorly defined and it is generally accepted that MS is caused by complex interactions between genetic and environmental factors [14]. Of note, signaling pathways such as NF-κB, JAK-STAT, and MAPK pathways in immune-regulating cells critically regulate inflammation in MS. Considering that ubiquitination is indispensable for signal transduction, functions of ubiquitin-modifying enzymes, particularly DUBs, are drawing increasing interests in this field. Indeed, mutations in several DUBs, such as USP18 and A20, have been found to be associated with MS [15–17]. Key characteristics of MS can be in part recapitulated by the mouse model experimental autoimmune encephalomyelitis (EAE) and it is therefore widely used to study the pathogenesis and treatment of MS [18]. Studies in EAE and MS have shown that DUBs regulate CNS autoimmune diseases by manipulating not only immune cells but also CNS-resident cells.

## DUBs regulate immune cells in MS/EAE

Infiltration of the CNS by T cells and other immune cells is a pathological hallmark of MS, and T cells have been identified as the critical mediators of both MS and EAE. However, CD8^+^ T cells outnumber CD4^+^ T cells in active MS lesions, whereas in EAE CD4^+^ T cells dominate over CD8^+^ T cells in demyelinating lesions [19, 20]. IFN-γ-producing T helper 1 (Th1) cells, IL-17-secreting Th17 cells, and GM-CSF-expressing T cells are key effector T cell subpopulations in EAE, and all of these T cell subsets can induce EAE independently [21, 22]. T cell responses are critically controlled by T cell receptor (TCR) signaling, which has been shown to be influenced by DUBs including USP9X [23], USP15 [24], and CYLD [25]. In CNS autoimmunity, the encephalitogenic property of T cells is dependent on the differentiation, activation, and survival of T cells, which are tightly regulated by ubiquitination and DUBs. Th17 cells are critical mediators of MS/EAE and are targeted for the treatment of MS [26]. Differentiation of Th17 cells is directed by the transcription factor RORγt, which also drives the production of GM-CSF, a decisive cytokine in EAE development [22]. RORγt protein levels are regulated partially by the UPS, and several DUBs, including USP4 [27] and USP17 [28], are required for RORγt stability. However, DUBA (OTUD5) promotes RORγt degradation indirectly by stabilizing the E3 ligase UBR5, which ubiquitinates RORγt for proteasomal degradation [29]. In addition to protein abundance, the activity of RORγt is also regulated by ubiquitination. Ubiquitination of RORγt at K446 impairs the interaction between RORγt and the coactivator SRC1, thereby inhibiting Th17 differentiation [30]. The K446 site-specific ubiquitination of RORγt can be stripped by USP15; therefore, USP15 functions as a positive regulator in Th17 differentiation [30]. The development, differentiation, and function of Th1 cells are controlled by the transcriptional factor T-bet [31]. Stability of T-bet has been shown to be positively regulated by USP10, which directly interacts with T-bet to mediate its deubiquitination [32]. IFN-γ is a finger print cytokine of Th1 cells, and it induces various immune responses including Th1 differentiation by activating the JAK-STAT1 signaling. IFN-γ-induced signaling is strongly enhanced by USP13, which exerts this function by deubiquitinating and stabilizing STAT1 [33]. Secretion of IFN-γ by Th1 cells is significantly increased in the absence of GRAIL, an E3 ligase that is crucial in the induction of CD4^+^ T cell anergy [34]. Prior oral feeding of myelin basic protein (MBP) ameliorates MBP-induced EAE. However, this oral tolerance is aborted by the ablation of GRAIL due to loss of T cell anergy [34]. The anergy-inducing function of GRAIL is safeguarded by USP8, which is essential for the deubiquitination and stability of GRAIL [35]. Thus, USP8 might be a potent regulator of T cell function and EAE.

**Fig. 1:**
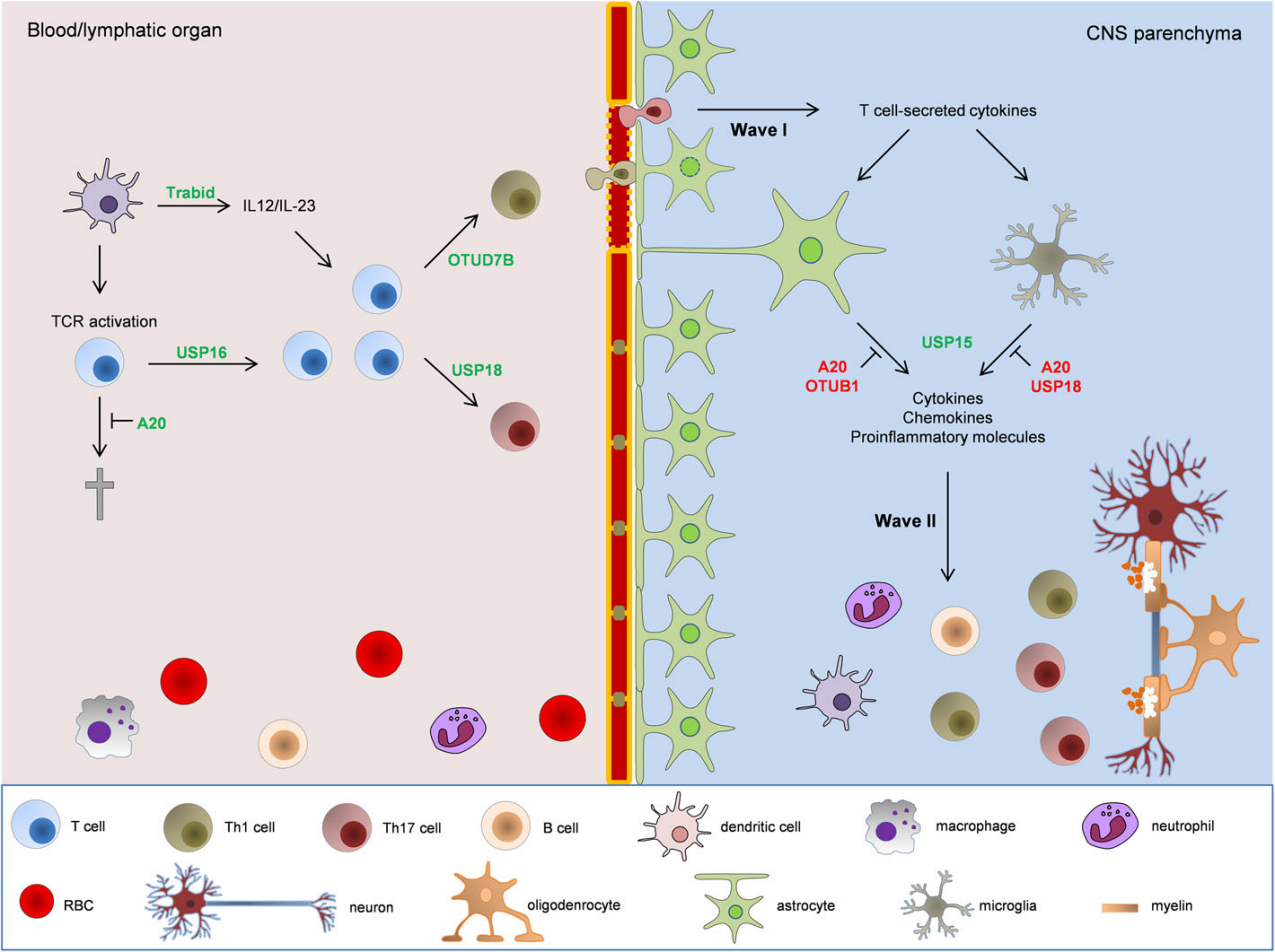
Role of DUBs in EAE. Upon EAE induction, T cells are primed in peripheral lymphatic organs and differentiate into autoreactive Th1 and Th17 cells. The priming, survival, and differentiation of T cells are enhanced by Trabid, USP16, USP18, A20, and OTUD7B. Before the clinical onset of EAE, only a few myelin-reactive T cells infiltrate the CNS (wave I). T cells in wave I are reactivated by APCs and produce cytokines including TNF, IL-17, and IFNs, which stimulate astrocytes and microglia. Activated astrocytes and microglia produce proinflammatory cytokines, chemokines, and molecules, which favor the establishment of an inflammatory environment and the massive recruitment of leukocytes (wave II), leading to clinical EAE onset and development. The wave I to wave II infiltration of leukocytes is switched by astrocytes and microglia, which are critically regulated by A20, OTUB1, USP15, and USP18. EAE-promoting DUBs are marked in green and EAE-inhibiting DUBs are marked in red

Although autoreactive T cells aggravate MS/EAE, neuroinflammation in these diseases can be strongly alleviated by regulatory T cells (Tregs). Differentiation of Tregs is tightly controlled by the transcription factor STAT5 [36], which has been shown to be positively regulated by the DUB POH1. Consistently, development of Tregs is severely impaired in T cell-specific POH1 knockout mice [37]. Furthermore, the immunosuppressive function of Tregs is negatively regulated by the Lys48-linked ubiquitination of Foxp3, which is mediated by the E3 ligase STUB1 [38]. The DUB USP7 deubiquitinates and stabilizes Foxp3 to positively regulate Treg functions [39]. The DUBs mentioned above are potential regulators of MS and EAE, but their roles have yet to be proven by further studies. So far, the function of OTUD7B, USP16, USP18, A20, and Trabid in immune cells has been elucidated in the pathogenesis and development of EAE (Fig. 1). Intriguingly, among the five DUBs, mutations in or close to human *USP18* and *TNFAIP3* (gene for A20) genes have been found to be associated with MS susceptibility [15–17]. The detailed functions of these DUBs will be discussed in subsequent subsections. It is noteworthy that most of the results were so far only obtained with the C57BL/6 EAE model. These studies provide an important conceptual framework on how neuroinflammation, CNS autoimmunity, and demyelination can be regulated but strictly need confirmation in MS patients, in particular due to the discrepancies between EAE and MS.

### OTUD7B

**Fig. 2:**
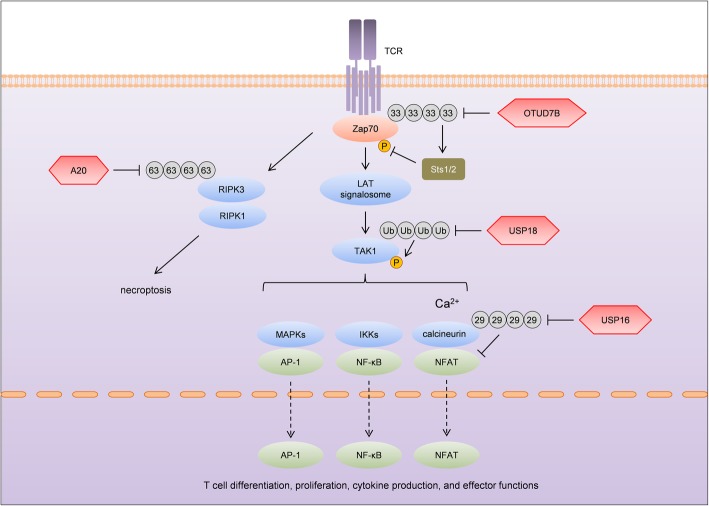
DUBs regulate EAE by modulating TCR signaling. T cells are essential effector cells in EAE and their functions are critically dependent on TCR-induced signaling, which is fine-tuned by DUBs. OTUD7B inhibits K33 ubiquitination of Zap70 and thereby potentiates its phosphorylation by reducing the recruitment of Zap70 inhibitors Sts1 and Sts2, leading to increased T cell activation and Th1 differentiation. USP18 deubiquitinates TAK1 and inhibits its activity, resulting in reduced NF-κB and NFAT activation and subsequent IL-2 production. Since IL-2 suppresses Th17 polarization, Th17 differentiation is strongly enhanced by USP18. Upon intracellular calcium stimulation, the binding of NFAT with calcineurin is inhibited by K29 ubiquitination of calcineurin, which can be stripped by USP16. By enhancing NFAT-mediated gene transcription, USP16 contributes to both the maintenance and proliferation of T cells. In addition to gene transcription, activation of TCR leads to necroptosis of T cells by inducing formation of the RIPK1-RIPK3 complex, which is positively regulated by K63 ubiquitination of RIPK3. By deubiquitinating RIPK3, A20 disrupts the RIPK1-RIPK3 complex and protects T cells from necroptosis

Hu et al*.* showed that OTUD7B, a member of the OTU subfamily, exacerbated EAE by positively regulating TCR signaling [40]. In response to TCR activation, Zap70, an essential signaling kinase in proximal TCR signaling, is both phosphorylated and ubiquitinated. Lys33-linked polyubiquitination of Zap70 signals recruitment of the phosphatases Sts1 and Sts2, which inhibit Zap70 phosphorylation and TCR signaling [41, 42]. As a versatile DUB, OTUD7B has been shown to cleave Lys11-, Lys33-, Lys48-, and Lys63-linked polyubiquitin chains [43–46]. Upon TCR stimulation, OTUD7B binds to and deubiquitinates Zap70, thereby preventing the binding of Sts1 and 2 [40]. Therefore, OTUD7B functions as a positive regulator of TCR signaling (Fig. 2). Deficiency of OTUD7B downregulates TCR-CD28-induced signaling, attenuates T cell activation, and impairs Th1 differentiation. Consistently, OTUD7B knockout mice are refractory to EAE [40].

### Trabid

Activation and differentiation of T cells are controlled by dendritic cells (DCs), which are professional antigen-presenting cells (APCs). In addition to their conventional role of priming T cells by antigen presentation, DCs can also activate CD8^+^ T cells by cross-presentation of exogenous antigens and produce T cell-regulating cytokines including IL-12 and IL-23 [47, 48]. The disease-promoting function of DCs in EAE is enhanced by Trabid (tumor necrosis factor receptor-associated factor-binding protein domain). The DUB Trabid, also called Zranb1, is a member of the OTU family that preferentially cleaves Lys29- and Lys33-linked polyubiquitin chains [49–51]. EAE was ameliorated in global and DC-specific Trabid knockout mice with reduced Th1 and Th17 differentiation [52]. Of note, Trabid has no cell-autonomous effect on T cell differentiation and EAE pathogenesis because these parameters are not altered by specific deletion of Trabid in T cells. Instead, Trabid regulates T cell differentiation by enhancing the production of IL-12 and IL-23 in DCs. IL-12 supports Th1 differentiation while IL-23, together with other cytokines, polarizes T cells to the Th17 subset. Noteworthy, IL-12 and IL-23 share a common p40 subunit that is encoded by the *Il-12b* gene [53]. Trabid is required for TLR-induced recruitment of c-Rel, binding of the RNA polymerase II complex, and most importantly, histone modifications, to the *Il-12b* promoter in DCs. Histone modification is positively regulated by the demethylase Jmjd2d, whose stability is critically regulated by Lys11- and Lys29-linked polyubiquitination [54]. As a Lys29-specific DUB, Trabid interacts with and stabilizes Jmjd2d by cleaving its Lys29-linked and to a less extent Lys11-linked polyubiquitin chains, thereby increasing active histone modification and concomitant *Il-12b* transcription [52]. Thus, EAE pathogenesis is positively regulated by Trabid, which functions by increasing the production of IL-12 and IL-23 in DCs.

### USP16

USP16 has been shown to regulate chromosome function by deubiquitinating PLK1 and histone H2A [55, 56]. Aberrantly increased expression of USP16 is associated with Down’s syndrome (DS) [57]. However, children with DS are predisposed to acute lymphoblastic leukemia (ALL), indicating that immune cells are possibly regulated by USP16 [58]. A recent study by Zhang et al. demonstrated that expression of USP16 is significantly increased in T cells of patients with autoimmune diseases [59]. Upon intracellular calcium stimulation, the Lys29 polyubiquitination of calcineurin A (CNA) blocks the recruitment of NFAT and this polyubiquitination can be reduced by USP16 (Fig. 2). Deletion of USP16 increases ubiquitination of CNA and thereby reduces transcription of NFAT-induced genes. As a result, both the maintenance and proliferation of T cells are defective in T cell-specific USP16 knockout mice, and these mice are refractory to T cell-mediated autoimmune diseases including inflammatory bowel disease and EAE [59]. Specific USP16 inhibitors might be of potential efficacy in treating autoimmune diseases mediated by T cells, such as MS.

### USP18

USP18 was first identified as a deubiquitinating enzyme in AML1-ETO leukemia [60]. In addition, USP18 is the only known protease that specifically deconjugates interferon-stimulated gene product 15 (ISG15), a ubiquitin-like protein, from target proteins [61]. ISG15 is conjugated to lysine residues of substrates in a process called ISGylation, which is catalyzed sequentially by an E1 activating enzyme (Ube1L), an E2 conjugating enzyme (UbcH8), and an E3 ligase (TRIM25 or Herc5) [62]. A broad range of cellular activities, especially type I IFN responses against viruses, are regulated by ISGylation. USP18 inhibits type I IFN signaling by stripping ISG15 from key signaling molecules such as STAT1. Independent of its catalytic activity, USP18 has been shown to inhibit type I IFN signaling by interaction with the type I IFN receptor-2 (IFNAR2) subunit of the IFNAR [63]. USP18 can increase the pool of unconjugated ISG15 by reversing ISGylation [64]. Unlike ubiquitin, free ISG15 acts as an extracellular cytokine. ISG15 binds to leukocyte function-associated antigen-1 (LFA-1) on T cells and NK cells and activates IFN-γ production [65]. Collectively, by reducing ISGylation and increasing free ISG15, USP18 plays important roles in immune responses such as anti-viral immunity. Polymorphisms related with the *USP18* gene were found to be associated with MS susceptibility and response to IFN-β treatment [15, 16]. In addition, haplotypic analysis identified one haplotype that is correlated with lower *USP18* gene expression in peripheral blood mononuclear cells and higher clinical symptoms [16]. The function of T cell-derived USP18 has been elucidated in EAE [66]. Upon TCR activation, USP18 interacts with TAK1 and reduces its ubiquitination (Fig. 2). Given that TAK1 ubiquitination is essential for its kinase activity, USP18 impairs NF-κB and NFAT activation and subsequent IL-2 production. Since IL-2 inhibits Th17 polarization and Th17 cells are essential for EAE pathogenesis [67], USP18-deficient T cells are defective in Th17 differentiation and EAE induction. This study shows that T cell-derived USP18 contributes to EAE pathogenesis [66]. However, the finding is in contrary to the clinical observation that lower *USP18* expression correlates with higher MS severity [16], indicating that mutations of *USP18* in other cell populations, rather than CD4^+^ T cells, might be responsible for MS pathogenesis. CD8^+^ T cells, B cells, and microglia, which have been shown to contribute to demyelination and neurodegeneration in MS [68], are top candidates.

### A20

A20, encoded by the *TNFAIP3* gene, is a critical gatekeeper of immune homeostasis. Unlike other DUBs, A20 contains both DUB and E3 ligase domains [69]. The N-terminal OTU domain confers A20 the DUB activity towards essential NF-κB signaling factors such as RIPK1, TRAF6, and NEMO [69–71]. In addition, A20 also possesses an E3 ligase activity, which is bestowed by the seven zinc finger (ZF) domains in the C-terminus. A20 inhibits RIPK1 in two sequential steps: Initially, as a DUB, A20 removes Lys63-linked polyubiquitin chains from RIPK1 to inactivate its activity. Subsequently, utilizing the E3 ligase function, A20 attaches Lys48-specific polyubiquitin chains on RIPK1 to induce its proteasomal degradation [69]. The double-safety mechanism of RIPK1 inhibition ensures the efficient blockade of the NF-κB cascade by A20. Mutations or polymorphisms in or close to the *TNFAIP3* gene have been linked to human autoimmune diseases including type I diabetes [72], psoriasis [73], systemic lupus erythematosus [74], systemic sclerosis [75], rheumatoid arthritis [76, 77], and MS [17]. *TNFAIP3* expression in blood was found to be inversely correlated with clinical features in Italian patients with relapsing-remitting MS [78]. Further investigation showed that *TNFAIP3* was downregulated in monocytes and CD4^+^ T cells of MS patients [79]. Consistent with clinical studies, investigations with mice have revealed that A20 functions in many cell types to maintain homeostasis and restrict autoimmunity. Unlike mice deficient for A20 specifically in DCs [80–82] or myeloid cells [83], which spontaneously develop autoimmunity, T cell-restricted A20 knockout mice develop normally and do not display spontaneous diseases [84], indicating that T cell-specific A20 is dispensable for immunological homeostasis. A20-deficient T cells are slightly more activated than A20-sufficient T cells. Surprisingly, most of the A20-deficient T cells die by 48 h after TCR stimulation, akin to activation-induced cell death. The death of A20-deficient T cells is not mediated by caspase-dependent apoptosis but mediated by RIPK3-dependent necroptosis. Mechanistically, utilizing its DUB activity, A20 reduces Lys63-linked ubiquitination of RIPK3 at Lys5, which is required for optimal induction of the RIPK1-RIPK3 complex and subsequent necroptosis (Fig. 2). Since A20 protects T cells from RIPK3-mediated necroptosis, T cell-specific A20-deficient mice have less activated T cells after immunization with MOG peptide and are partially protected from EAE [84].

## DUBs regulate CNS-resident cells in MS/EAE

Both CNS homeostasis and neuroinflammation can be to a large extent controlled by CNS-resident cells such as microglia, a CNS-resident macrophage-like cell population, and astrocytes, the most abundant cells in the CNS. Microglia and astrocytes are emerging as crucial regulators of CNS autoimmunity, and their multi-faceted functions in MS and EAE have been summarized by other reviews [85, 86]. The immune-regulating abilities of microglia and astrocytes are primarily ascribed to their responsiveness to cytokines secreted by infiltrating encephalitogenic T cells. Since the cytokine-induced signaling pathways are closely modulated by the ubiquitination system, DUBs serve as fine-tuning regulators of microglia and astrocytes. Thus far, OTUB1, USP15, USP18, and A20 have been identified as crucial regulators of microglia or astrocytes in EAE (Fig. 1).

### OTUB1

**Fig. 3:**
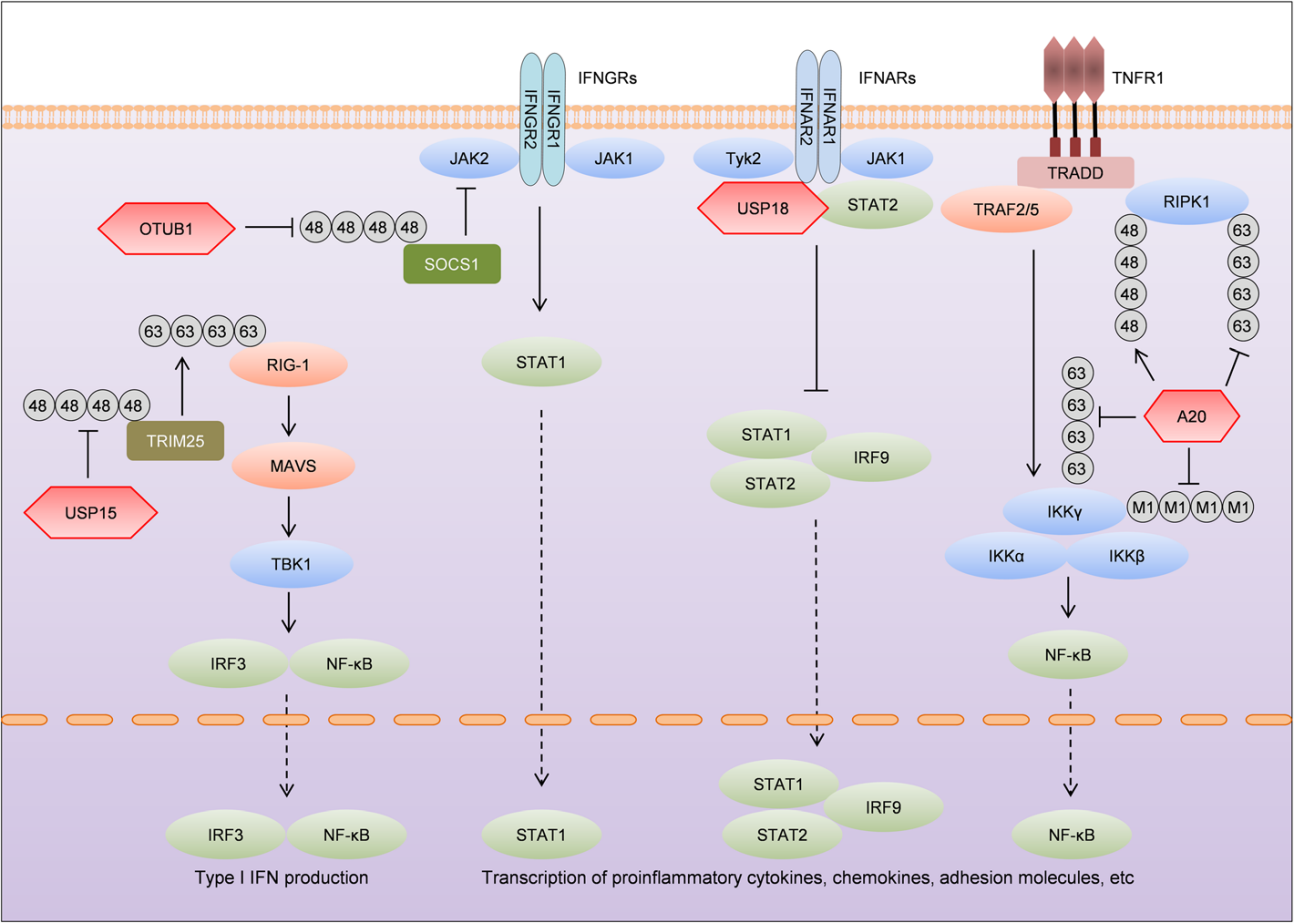
DUBs regulate EAE by modulating signal transduction in astrocytes and microglia. In addition to immune cells, the onset and perpetuation of EAE can also be influenced by astrocytes and microglia, two CNS-resident cell populations with immune-regulating properties. Production of type I IFNs is induced by RIG-1-initiated signaling, and the activity of RIG-1 is enhanced by TRIM25, which mediates K63 ubiquitination of RIG-1. By deubiquitinating and stabilizing TRIM25, USP15 enhances RIG-1-dependent type I IFN production. Microglia activation induced by type I IFNs is detrimental for EAE, but it can be inhibited by USP18, which directly interacts with IFNAR2 and inhibits downstream signaling. IFN-γ, the type II IFN, induces the production of multiple cytokines and chemokines in astrocytes. IFN-γ-induced astrocyte activation is inhibited by OTUB1, which K48 deubiquitinates and stabilizes SOCS1, an inhibitor of JAK2. In addition to OTUB1, IFN-γ-induced astrocyte activation can also be inhibited by A20, which inhibits STAT1 transcription. A20 is a versatile ubiquitin-modifying enzyme and it processes both DUB and E3 ligase activities. Upon TNF stimulation, A20 catalyzes K48 ubiquitination of RIPK1 after removing K63 ubiquitin chains from it. Apart from RIPK1, activity of IKKγ is also inhibited by A20, which reduces M1 and K63 ubiquitination of IKKγ

OTUB1 is an OTU family DUB that has a preference for cleaving Lys48-linked polyubiquitin chains [87]. Unlike other DUBs, OTUB1 possesses not only a canonical DUB activity, which removes Lys48-linked polyubiquitin chains directly from substrates, but also a non-canonical DUB activity, which inhibits the transfer of Lys48- or Lys63-specific ubiquitin from E2 conjugating enzymes to E3 ligases, thereby impeding ubiquitination of target proteins [88, 89]. The canonical DUB activity of OTUB1 is required to stabilize c-IAP [90] and Tau [91], and the non-canonical activity is essential for deubiquitinating SMAD3 [92], chromatin [88], and p53 [93]. Given that OTUB1 is versatile in regulating key molecules involved in various cellular activities, it is not surprising that global OTUB1 knockout mice are embryonic lethal [94, 95]. A study by Zhou et al. shows that OTUB1 inhibits the activation of T cells and NK cells by dampening IL-15R signaling [96]. Given that T cells and NK cells are key mediators in MS [97], OTUB1 might influence EAE by regulating the functions of T cells and NK cells. Mice deficient of OTUB1 selectively in astrocytes do not show abnormalities under physiological conditions but develop more severe EAE [95]. Of note, astrocytes play both protective and detrimental roles in EAE [86]. However, according to the two-wave theory of EAE development, astrocytes contribute to EAE, at least in the early stage, by producing leukocyte-recruiting molecules [98]. After priming in peripheral lymphatic organs, only a few myelin-reactive CD4^+^ T cells traffic through choroid plexus or leptomeningeal vessels to the subarachnoid/perivascular space (wave I), where they are reactivated by APCs [99]. As a result, CD4^+^ T cells expand and produce cytokines including TNF, IFN-γ (by Th1 cells), and IL-17 (by Th17 cells). These cytokines stimulate adjacent CNS-resident cells, particularly astrocytes, to produce leukocyte-recruiting cytokines and chemokines, which amplify the infiltration of inflammatory cells to the CNS in a positive-feedback loop (wave II), leading to clinical EAE onset [100]. Astrocyte-specific OTUB1 inhibits the conversion of wave I to wave II by suppressing proinflammatory gene production in astrocytes induced by IFN-γ [95]. Mechanistically, OTUB1 inhibits the IFN-γ-activated JAK2-STAT1 signaling by Lys48 deubiquitinating and stabilizing SOCS1, the inhibitor of JAK2 (Fig. 3). In humans, OTUB1 is highly expressed in astrocytes of MS patients, implying that astrocytic OTUB1 might be involved in the pathogenesis and development of MS [95].

### USP15

Structurally, USP15 contains a DUSP (domain in ubiquitin-specific protease) domain, two ubiquitin-like folds, and a large catalytic domain [101, 102]. Torre et al. identified a mutation in the gene encoding USP15 (USP15^L749R^) that protected mice from EAE and experimental cerebral malaria [103]. L749R substitution acts as a loss of function of USP15, and USP15 protein is not detectable in USP15^L749R^ mice. Upon active EAE induction with MOG peptide, USP15^L749R^ mice show reduced clinical symptoms and mortality in comparison with C57BL/6 control mice [103]. Global RNA-sequencing analysis of spinal cord tissue during EAE suggests that USP15 is involved in type I IFN response, which induces cytokines and chemokines that recruit leukocytes to the site of inflammation. USP15 interacts with and deubiquitinates TRIM25, an E3 ligase that positively regulates RIG-1-mediated production of type I IFNs, to prevent its degradation (Fig. 3). The USP15 L749R mutant dampens the type I IFN response due to its impotence to deubiquitinate TRIM25, thereby attenuating EAE. USP15 is expressed in human microglia and astrocytes at steady state and its levels in these two cell populations can be further induced by cytokines such as IFN-γ, implying that IFN-induced activation of these cells might be regulated by USP15. The study by Torre et al. shows that USP15 is required for the pathogenesis of EAE [103]. However, further investigations with tissue-specific knockout mice are required to identify the cell types that contribute to EAE by USP15.

### USP18

In addition to T cell-specific USP18, the function of microglia-derived USP18 has also been explored. In mice, USP18 is identified as a regulatory molecule that prevents aberrant activation of microglia [104]. USP18 is highly expressed in unstimulated microglia and inhibits activation of microglia induced by type I IFN, which is constitutively expressed in some tissues including the CNS. The USP18-mediated inhibition of type I IFN signaling is independent of its catalytic activity but requires the physical interaction with IFNAR2 (Fig. 3). Already under physiological conditions, USP18-deficient microglia in the white matter of both global (USP18^−/−^) and myeloid-specific (CX3CR1-Cre USP18^fl/fl^) USP18 knockout mice show signs of activation. Upon EAE induction, CX3CR1-Cre USP18^fl/fl^ mice display significantly more severe EAE symptoms in the effector phase of the disease with increased lymphocyte infiltration and axonal damage in the upper white matter of the CNS [104]. Therefore, USP18 not only preserves microglia homeostasis but also prevents overshooting neuroinflammation in CNS autoimmunity.

### A20

Unlike global A20 knockout mice which develop spontaneous neuroinflammation [105], mice with targeted ablation of A20 in neuroectodermal cells including astrocytes, neurons, and oligodendrocytes or specifically in astrocytes do not show obvious CNS defects. However, ablation of A20 in astrocytes aggravates EAE [106]. A20 is found to inhibit cytokine and chemokine production in astrocytes by suppressing NF-κB and JAK-STAT signaling pathways (Fig. 3). In addition to astrocytes, functions of microglia are also critically regulated by A20 in EAE [107]. A20 is found to inhibit both the priming and activation of NLRP3 inflammasome in microglia. Consistently, deletion of A20 specifically in microglia renders mice hypersensitive to EAE due to increased proinflammatory gene production resulting from augmented activation of the NLRP3 inflammasome [107].

## Opposite roles of DUBs in immune cells and CNS-resident cells

Genome-wide association studies (GWASs) of MS patients revealed that polymorphisms in or close to human *TNFAIP3* and *USP18* genes are associated with MS susceptibility [16, 17]. Therefore, the DUBs A20 and USP18 might be potential targets for MS treatment. Studies with tissue-specific mice showed that T cell-specific A20 and USP18 exacerbate EAE via enhancing survival (A20) and activation (USP18) of T cells [66, 84]. Based on these studies in the C57BL/6 EAE model, polymorphisms associated with reduced expression or loss of function of A20 and USP18 would be expected to decrease MS risk and severity. In sharp contrast, lower expression of *TNFAIP3* and *USP18* in blood has been found to be correlated with more severe MS [16, 79]. This incongruence indicates that SNPs in *TNFAIP3* and *USP18* in T cells might not contribute to MS onset and/or progression. Of note, the reduced *TNFAIP3* and *USP18* levels in MS blood might be caused by changes in T cell subsets rather than direct regulation of these genes in a static T cell population. Here, it is also important to keep in mind that the pathogenesis of MS and EAE shows several differences with respect to the relative importance of T cell subsets and B cells [68]. Astrocyte-specific A20 and microglia-specific A20 or USP18 knockout mice develop more severe EAE [104, 106, 107], and, thus, these studies may provide biological rationale for the clinical observations. Confirmation of a reduced or lacking A20 and USP18 expression in microglia/astrocytes in MS lesions might open the way for novel treatment strategies for MS by targeting CNS-resident DUBs. In addition, the total opposite effects of CNS-resident cell- and T cell-derived USP18 and A20 in EAE stress the importance of the tissue-specific study of DUBs.

## Conclusions

Immune responses in CNS autoimmunity are tightly regulated by ubiquitination, which is fine-tuned by DUBs. DUBs are drawing increasing interest for the treatment of various diseases such as cancer and autoimmune diseases [108]. On one hand, blocking one or several cytokines for the treatment of MS is usually insufficient considering the complex nature of this disease; on the other hand, blocking certain signaling pathways, such as the NF-κB pathway, might cause severe complications given that these signaling pathways are vital for maintaining normal cellular functions. Specific and potent DUB inhibitors or agonists might not only provide adequate efficacy for mitigating neuroinflammation in MS but also circumvent the problem of provoking vicious complications. However, more data are required on the expression of DUBs in immune cells (B cells, T cells, and macrophages), ideally from blood and CSF, as well as brain-resident cells in MS lesions. This may also lead to the identification of novel DUBs and the molecular mechanism of DUB function, allowing the development of specific DUB inhibitors/agonists for the treatment of this CNS autoimmune disease.

## Data Availability

Not applicable.

## Abbreviations

ALL: Acute lymphoblastic leukemia
APC: Antigen-presenting cell
CNA: Calcineurin A
CNS: Central nervous system
DC: Dendritic cell
DS: Down’s syndrome
DUB: Deubiquitinating enzyme
DUSP: Domain in ubiquitin-specific protease
EAE: Experimental autoimmune encephalomyelitis
GWAS: Genome-wide association study
IFNAR: Type I IFN receptor
ISG15: Interferon-stimulated gene product 15
JAMM: JAB1/MPN/MOV34 metalloprotease
LFA-1: Leukocyte function-associated antigen-1
MBP: Myelin basic protein
MINDY: Motif interacting with ubiquitin (MIU)-containing novel DUB family
MS: Multiple sclerosis
OTU: Ovarian tumor protease
PTM: Post-translational modification
TCR: T cell receptor
Trabid: Tumor necrosis factor receptor-associated factor-binding protein domain
Treg: Regulatory T cell
UCH: Ubiquitin C-terminal hydrolase
UPS: Ubiquitin-proteasome system
USP: Ubiquitin-specific protease
ZF: Zinc finger
